# Left subclavian artery malperfusion due to thoracic outlet syndrome during total vertebrectomy for invasive lung cancer: a case report

**DOI:** 10.1186/s40981-017-0131-4

**Published:** 2017-11-25

**Authors:** Kazuyuki Mizunoya, Kentaro Ueda, Yoshifumi Takeda, Koichi Takita, Yuji Morimoto

**Affiliations:** Department of Anesthesiology, Hokkaido University Hospital, N15 W7 Kita-ku, Sapporo, 0608638 Japan

**Keywords:** Artery malperfusion, Interarm pressure difference, Thoracic outlet syndrome, Prone position, Total vertebrectomy

## Abstract

Thoracic outlet syndrome (TOS) can interrupt blood flow to upper limbs by vascular compression. We report a case of a 52-year-old man who presented left subclavian artery malperfusion due to TOS during total vertebrectomy (Th2–4) in the prone position for invasive lung cancer. At the time of resection of the vertebral bodies, his left radial systolic blood pressure had begun to drop intermittently and we noticed an interarm pressure difference. Accordingly, we began to monitor the right radial artery pressure and found that only the left radial artery pressure decreased as a result of compressive force from the surgical site. The operation was continued with intermittent malperfusion of the left arm, and when it was prolonged, we asked the surgeons to release the compression. No symptoms of ischemia or nerve injuries in the left arm were observed after the surgery. Retrospective review of his preoperative enhanced computed tomography images suggested a slightly compressed left subclavian artery in the costoclavicular space. Combination of the prone position and a specific upper limb position may be a risk factor for intraoperative TOS. An interarm blood pressure difference is a clue to detect accidental arterial TOS during general anesthesia.

## Background

Thoracic outlet syndrome (TOS) is a condition that occurs when the neurovascular bundle is compressed in the thoracic outlet space formed by the first rib, the clavicle, and the scalene muscles. It has many causes such as congenital anomalies and traumatic injuries. There are three different subtypes of TOS: neurogenic, arterial, and venous according to the predominantly affected structures [1]. The occurrence of TOS under general anesthesia is considered rare and difficult to recognize, so only a few case reports have been published [2–4].

We report a case of left subclavian artery malperfusion suspected to be caused by TOS during thoracic total vertebrectomy in the prone position for invasive lung cancer.

## Case presentation

A 52-year-old man was found to have pulmonary adenocarcinoma of the right upper lobe with invasion to upper thoracic vertebral bodies. Complete tumor resection was planned via right upper lobectomy and total vertebrectomy (Th2–4) with corresponding costectomy. Prior to surgery, catheter embolization was tried to prevent massive bleeding from the tumor and around the vertebrae, but it failed. Accordingly, total vertebrectomy following bilateral video-assisted thoracoscopic surgery was scheduled for ligation of feeding intercostal arteries and exfoliation of the tumor.

After administration of 100% oxygen, general anesthesia was induced with propofol, fentanyl, remifentanil, and rocuronium. For maintenance of anesthesia, we used propofol and remifentanil in consideration of motor evoked potential monitoring. An arterial line was placed in the left radial artery and a pulse oximeter attached to his left hand.

**Fig. 1:**
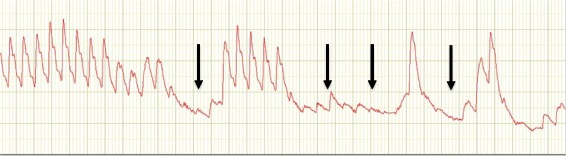
Left radial artery pressure waveform. Pulse pressure was highly reduced and disappeared intermittently (black arrows)

The intrathoracic procedure in the right and left lateral decubitus positions was completed uneventfully. Then, the patient was turned to the prone position for total vertebrectomy approached via a posterior midline incision. When the surgical procedure reached the area around the vertebral bodies, arterial systolic blood pressure suddenly dropped from 90 to 43 mmHg. Because we suspected massive hemorrhage from vessels around the vertebrae, we asked the surgeons to interrupt the operation. At the same time, we performed rapid infusion and transfusion and administered phenylephrine. Then, his blood pressure was restored to the previous value and the operation was resumed. After a short time, his blood pressure dropped again (Fig. 1) without any sign of bleeding at the surgical site, so we considered other reasons for the low blood pressure. Despite extremely low pulse pressure in the left radial artery, we could palpate the right radial artery, so we started to monitor the pressure of the right radial artery. We found an interarm pressure difference and the possibility of reduced blood flow in the left upper limb. We suspected left subclavian artery malperfusion due to the surgical procedure at that time. We were also afraid of malperfusion of an unmonitored aortic arch branch, the left common carotid artery, because we thought that resection of intercostal arteries and exfoliation between the upper thoracic vertebral bodies and posterior mediastinum tissues caused impaired fixation of the descending aorta and aortic arch, which might easily result in ventral displacement under the influence of pressure from the dorsal side. We considered whether to add a cerebral regional oxygenation (rSO_2_) monitoring, but attachment of rSO_2_ sensors to his forehead was difficult in the prone position. The bispectral index and motor evoked potentials of the upper extremities were unchanged. Therefore, the operation continued with intermittent malperfusion of the left arm, and when it was prolonged, we asked the surgeons to release the compression. The malperfusion was observed over 10 h until closure of the thoracic cavity and never observed after that. The total time of operation was 23 h (the prone position lasted over 17 h) and intraoperative blood loss was 4220 ml.

The patient was transported to our ICU under mechanical ventilation and extubated 30 h after his ICU admission. He did not have any signs of cerebral ischemia or left arm neurological or ischemic damage.

**Fig. 2:**
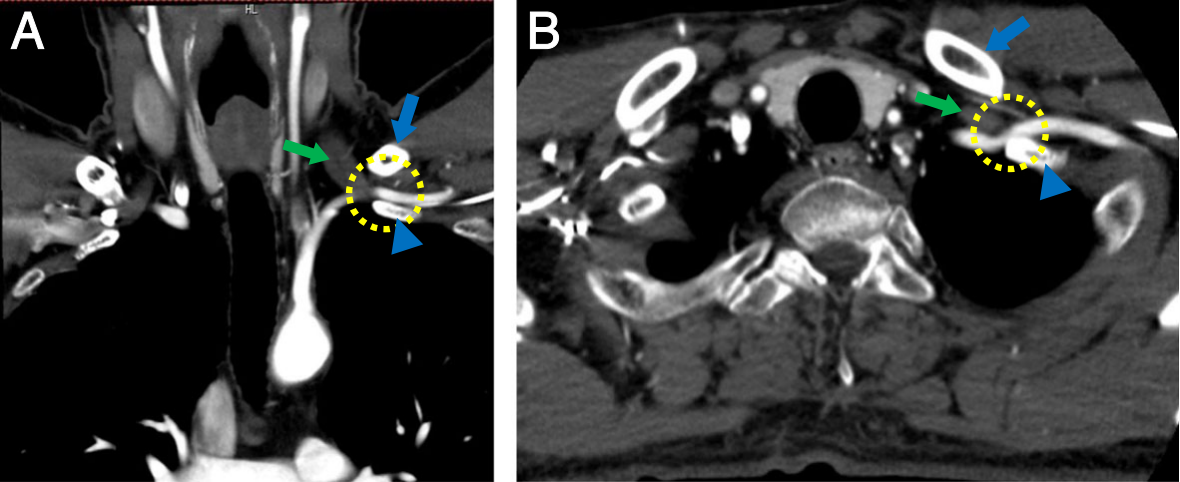
Preoperative enhanced CT (**a** coronal view, **b** axial view). Yellow dotted circles indicate the slightly compressed left subclavian artery in the left costoclavicular space. (blue arrow: clavicle, blue triangle: the first rib, green arrow: anterior scalene muscle)

Postoperatively, we reviewed enhanced computed tomography scans of the patient, and a slightly compressed left subclavian artery between the first rib and anterior scalene muscle was pointed out (Fig. 2). However, he had never felt any clinical symptoms previously. His condition was finally diagnosed as accidental arterial TOS resulting from the surgical procedure and his position during the operation. Further diagnostic test such as CT angiography was not performed because of its little value for his postoperative treatment.

### Discussion

TOS is a disorder with signs and symptoms produced by compression of nerves or vessels, or both, at the thoracic outlet. The most common type is neurogenic TOS, and vascular types (arterial or venous) are relatively rare [1]. For anesthetized patients, TOS is a very critical condition because they cannot complain about their symptoms. Vascular types of TOS may be recognized as an interarm blood pressure difference, loss of the plethysmographic waveform, edema, and obstruction of IV lines in the affected limb, but neurogenic TOS cannot be noticed during general anesthesia.

There may be two major causes for the intraoperative occurrence of TOS, the position of the patient and compression of the thorax mainly due to surgical maneuvers. Mouton et al. [3] reported a case of positional arterial TOS in the left lateral park bench position for neurosurgery, and they continued the operation after repositioning of the patient. Kim et al. [4] reported a case of arterial TOS that occurred due to pressure applied to the surgical site during spine surgery in the prone position. In that position, the thorax tends to be compressed by the patient’s own weight and TOS symptoms seem likely to appear with compressive force from the dorsal side.

In our case, in addition to the prone position, the patient’s upper limb positioning might have affected the occurrence of TOS. Our patient’s upper limbs were fixed with shoulders in the 90° abducted position and elbows flexed to 90°, as for the Roos test, a diagnostic stress test for TOS [5]. This arm positioning stretches the neurovascular bundle and can provoke TOS symptoms. If we had noticed TOS and its mechanism, we could reposition his upper limbs to avoid malperfusion. The compression from the surgical site, together with positional risk factors, was considered to be the cause of the left subclavian artery malperfusion. We assumed that the ascending aorta and aortic arch were pressed and displaced ventrally during resection of the vertebrae and contributed to malperfusion in the current case. This is because the left radial artery pressure decreased even while surgeons did not compress his thorax during exfoliation between the upper thoracic vertebral bodies and posterior mediastinum tissues.

Because the arterial TOS in our case was not continuous but intermittent, we continued the operation after we noticed the malperfusion of the left subclavian artery. However, even if arterial compression is intermittent, there is a risk of intimal damage, aneurysm formation, and thrombosis due to repeated compression of the artery. If arterial TOS is accompanied by neurogenic TOS, severe peripheral nerve injuries may arise silently during the long period of general anesthesia. Accordingly, preoperative risk assessment based on the patient’s anatomical factors, positional risk during the operation, and surgical confounding is very important, not to mention early detection once it happens. For prevention of intraoperative TOS, it might be better to fix patient’s upper limbs along the trunk, if the patient has some risk factors of intraoperative TOS. For early detection of pulselessness in one side of an upper limb, it might be useful to monitor pulse waveform of bilateral upper limbs using pulse oximeters and/or arterial lines. Once TOS is suspected, careful repositioning of the patient may be effective to reduce compression of the neurovascular bundle.

## Conclusions

We experienced left subclavian artery malperfusion due to TOS during general anesthesia. Combination of the prone position and a specific upper limb position may be a risk factor for intraoperative TOS, even though patients have never felt any symptoms. An interarm blood pressure difference and pulse waveform laterality are clues to detect accidental arterial TOS during general anesthesia. Once it happens, careful repositioning of the patient may reduce TOS symptoms.

## Abbreviations

CT: Computed tomography
TOS: Thoracic outlet syndrome
